# *Tinospora cordifolia* protects against inflammation associated anemia by modulating inflammatory cytokines and hepcidin expression in male Wistar rats

**DOI:** 10.1038/s41598-019-47458-0

**Published:** 2019-07-29

**Authors:** Niraj S. Ghatpande, Ashwini V. Misar, Ravindra J. Waghole, Sachin H. Jadhav, Prasad P. Kulkarni

**Affiliations:** 1 Bioprospecting Group, Agharkar Research Institute, G. G. Agarkar Road, Pune, 411004 Maharashtra India; 2 Savitribai Phule Pune University, Ganeshkhind, Pune, 411007 Maharashtra India; 3 Animal House, Developmental Biology Group, Agharkar Research Institute, G. G. Agarkar Road, Pune, 411004 Maharashtra India

**Keywords:** Anaemia, Iron

## Abstract

Systemic iron homeostasis dysregulation is primarily associated with inflammation- associated anemia (AI) due to hepcidin up-regulation. *Tinospora cordifolia* (TC) has shown remarkable anti-inflammatory properties and has been found useful in the treatment of inflammatory disorders. However, the effects and mechanisms of TC on AI have not been studied yet. We conducted *in vivo* and *in vitro* studies to evaluate the effect of TC on AI. HPLC studies were also carried out to find out active constituents in TC extract. Model system exhibiting AI was developed by repeated injections of HKBA in Wistar rats. TC treated groups showed significantly higher levels of Hb and RBC count compared to the inflammatory control group. TC treatment showed reduction in the expression of the HAMP (hepcidin) gene in the rat liver. TC extract also inhibited gene expression of inflammatory cytokines (TNF-α, IL-1β) and decreased NO production in RAW 264.7 cells. The HPLC analysis revealed the presence of tinosporaside, which could have synergistically contributed to the above findings. Overall results indicate that TC therapy was able to maintain circulating iron through reduction of inflammatory cytokines and expression of hepcidin in rats.

## Introduction

Infection or inflammation associated anemia (AI) is the second most common form of anemia after iron deficiency anemia. Our previous study showed the synergistic association between inflammation, hepcidin, and anemia among adolescent girls^1^. AI is characterized by mild to moderate and normal anemia, the reduced lifespan of erythrocytes and suppressed erythropoiesis^2^. Perhaps the most characteristic feature of AI is the dysregulation of systemic iron homeostasis shown by hypoferremia despite intact tissue iron stores^2^. Hepcidin contributes in dysregulation of iron homeostasis. Under inflammatory conditions, hepcidin inhibits absorption of iron into enterocytes and the iron efflux into the circulation from reticuloendothelial macrophages^3^. AI therapy includes intravenous iron supplementation, blood transfusion, or erythropoietin treatment^2^. Recently, hepcidin agonists, as well as antagonists, are being developed for the treatment of AI^4^. These anti-hepcidin therapies are effective, however, an increased risk of infection or suppression of immune response is associated with these therapies^5^. Therefore, effective AI therapy must demonstrate effectiveness, with minimum side effects, in both acute and long-term settings.

In recent years, use of plants as medicine and food has become more important as good alternatives to synthetic medicines. In Indian traditional medicinal system, TC has been used for anti-inflammatory and immuno-modulatory purposes^6^. Phytochemical analysis of *Tinospora cordifolia* (TC) revealed that it contains alkaloids, glycosides, sesquiterpenoids, lactones, and steroids^7^. So far studies have been carried out to evaluate the anti-inflammatory and immuno-modulatory properties of TC^8,9^. The effect of TC against AI has not been studied yet and therefore we evaluated the effectiveness of TC for the treatment of AI.

## Results

### Acute oral toxicity

The rats treated with the TC at a dose of 2000 mg/kg of body weight showed no mortality or any untoward symptoms or abnormal behavioral changes during the 14 day observation period following dosing. The rats treated with the TC extract exhibited a normal pattern of body weight gain during the 14 days period. No changes were found in hematological and biochemical parameters due to treatment with the TC extract (Table 1). Thus TC extract was found to be non-toxic at a dose of 2000 mg/kg of body weight.

**Table 1:** Hematological and biochemical parameters of rats during the acute oral toxicity study.

Parameters	14^th^ day	
	Control	Experimental
Body Weight (g)	249.60 ± 22.02	254.27 ± 14.05
Hb (g/dL)	15.9 ± 0.4	15.3 ± 2.5
RBC (million/µL)	8.9 ± 0.30	7.60 ± 0.70
SGPT (IU/L)	17.50 ± 1.00	19.57 ± 2.00
Creatinine (mg/dL)	0.27 ± 0.03	0.36 ± 0.10

SGPT: Serum glutamic pyruvic transaminase.

### Hb levels and total RBC count in rats

**Figure 1:**
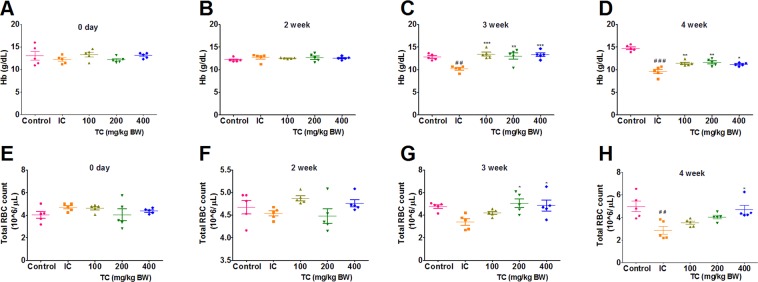
Effect on Hb levels (**A**–**D**) and RBC count (**E**–**H**) in rats treated/untreated with TC. IC: Inflammatory control, 100: 100 mg/kg, 200: 200 mg/kg and 400: 400 mg/kg of body weight of TC. Number of animals per group = 5. Results are shown as mean ± SEM. *p < 0.05, **p < 0.01 and ***p < 0.001 against the IC group and ^##^*p* < 0.01 and ^###^*p* < 0.001 against control.

Before induction of AI, we found no significant difference in the levels of Hb and RBC count in NC and TC treated rats (Fig. 1A,B,E,F). Injections of HKBA caused progressive decrease in Hb levels of rats in IC group compared to control rats at 3^rd^ week as shown in Fig. 1C. These Hb levels were further reduced and reached up to 9 g/dL in rats at 4^th^ week of study (Fig. 1D).

Commensurate with the decrease in Hb levels, we observed significantly lower RBC count in the HKBA injected rats compared to the control rats at 3^rd^ and 4^th^ week indicating anemic conditions. In case of rats treated with TC, the Hb levels were significantly higher compared to IC rats at 3^rd^ week (Fig. 1C). The Hb levels were slightly lower at 4^th^ week in TC treated group compared to controls; however, the Hb levels were still significantly higher than IC group. Similarly, the RBC count of rats treated with TC at 3^rd^ and 4^th^ week was higher than the RBC count of IC group (Fig. 1G,H). These observations indicate that treatment with TC significantly retarded induction of anemia due to the injections of HKBA.

### Serum and tissues iron levels

**Figure 2:**
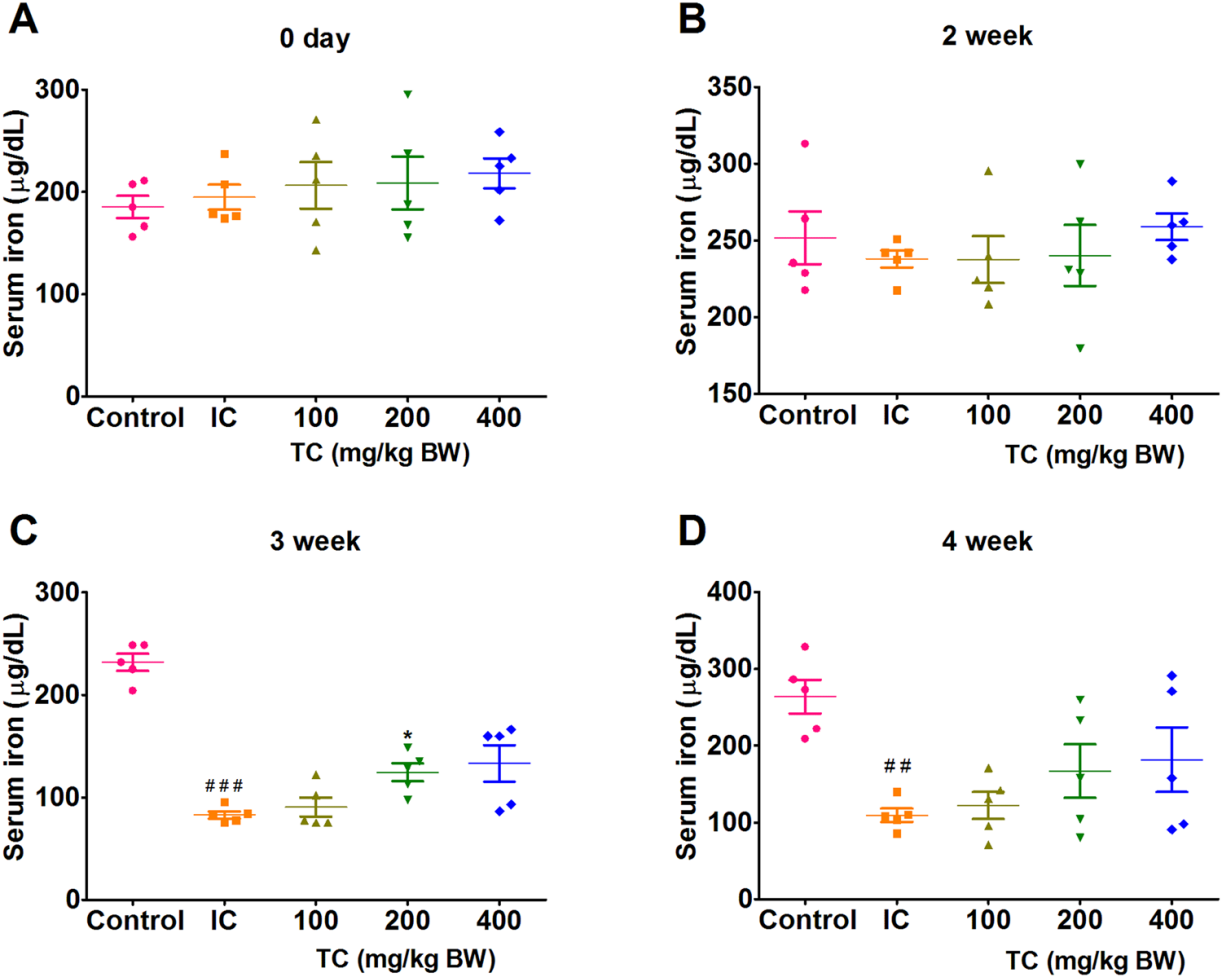
Changes in serum iron levels in rats treated groups. IC: Inflammatory control, 100: 100 mg/kg, 200: 200 mg/kg, and 400: 400 mg/kg of body weight of TC. Number of animals per group = 5. Results are shown as mean ± SEM. *p < 0.05, against the IC group and ^##^*p* < 0.01 and ^###^*p* < 0.001 against control.

We observed no significant difference in serum iron levels among IC, NC and TC treated group rats at 0 day and 2^nd^ week, i.e. prior to AI induction (Fig. 2A,B). Serum iron levels in IC group rats were found to be significantly lower than NC group rats at 3^rd^ and 4^th^ week (Fig. 2C,D). However, rats treated with TC showed higher serum iron levels compared to IC group in dose-dependent manner at 3^rd^ and 4^th^ week (Fig. 2C).

**Figure 3:**
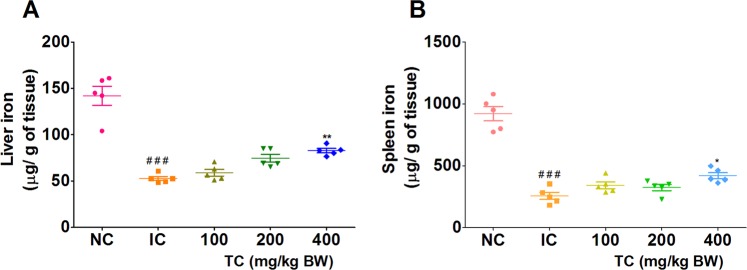
Effects of TC on liver and spleen iron levels. IC: Inflammatory control, 100: 100 mg/kg, 200: 200 mg/kg, and 400: 400 mg/kg of body weight of TC. Number of animals per group = 5. Results are shown as mean ± SEM. *p < 0.05 and **p < 0.01 against the IC group and ^##^*p* < 0.01 and ^###^*p* < 0.001 against control.

HKBA injections caused a significant reduction in liver and spleen iron stores as compared to control rats as shown in Fig. 3A,B. Treatment with TC at a dose of 400 mg/kg body weight showed significantly higher liver and spleen iron content (Fig. 3A,B). As at 4^th^ week iron levels were higher, therefore, the RBC count and Hb levels were maintained in rats treated with TC compared to the IC group.

### Body and organs weights

Body and organs weights can be the most sensitive indicator of chronic inflammation or infection. As shown in Table 2, repeated injections of HKBA in rats caused significant loss of body weight as well as in the weight of the liver. Further, we also observed enlargement of spleen size and weight i.e. splenomegaly in IC group. Rats treated with TC at a dose of 400 mg/kg body weight showed significant preservation of body weight and also the weight of liver and spleen (Table 2).

**Table 2:** Changes in body weight and weight of liver and spleen weight of rats in different groups.

S.N.	Group	Body weight (g)	Liver weight (g)	Spleen weight (g)
1	NC	314.46 ± 8.13	13.93 ± 0.86	0.86 ± 0.077
2	IC	239.93 ± 4.59^#^	8.84 ± 0.82^#^	3.23 ± 0.43^#^
3	TC: 100 mg/Kg body weight	249.16 ± 1.65	9.79 ± 0.89	3.33 ± 0.27
4	TC: 200 mg/Kg body weight	248.18 ± 9.03	10.09 ± 0.38^*^	2.70 ± 0.40
5	TC: 400 mg/Kg body weight	254.66 ± 9.89^*^	10.12 ± 0.21^*^	1.87 ± 0.12^*^

IC: Inflammatory control. Results are shown as mean ± SEM. Statistical analyses were carried out by using one-way ANOVA and Bonferroni *post-hoc* test with *p < 0.05, **p < 0.01 and ***p < 0.001 against the IC group and ^#^p < 0.05, ^##^p < 0.01 and ^###^p < 0.001 against control.

### Tissues histology

**Figure 4:**
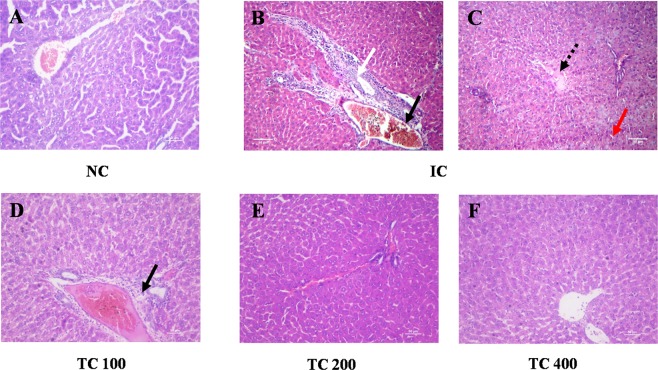
TC protects infection-induced liver damage. (**A**–**F**) Slides were observed under a light microscope at 40× magnification. In the figure white arrow shows the dilation of the bile duct, black arrow shows the infiltration of inflammatory cells, dashed arrow shows the necrosis, and red arrow shows tissue swelling and loss of tissue architecture. NC: Normal control, IC: Inflammatory control, TC 100: 100 mg/kg, TC 200: 200 mg/kg, and TC 400: 400 mg/kg of body weight of TC aqueous extract. Number of animals per group = 5. Scale bar = 50 µm.

Chronic inflammation is always associated with structural and cellular changes at the tissue level. Hence, histological examination of the liver and spleen was carried out to understand the effect against AI in rats treated with TC at the tissue level. Liver tissue from the NC group displayed normal cellular architecture, and also a lobular pattern with prominent central vein and intact sinusoids were evident (Fig. 4A). Liver tissue from IC group showed moderate to severe histological changes such as cellular swelling, inflammatory cells infiltration, vacuolar changes in the cytoplasm of hepatocytes, severe necrotic changes in hepatocyte and loss of nuclei. Moreover, we also found a heavy infiltration of mononuclear cells, microgranuloma, lymphoid aggregation, and moderate bile duct hyperplasia (Fig. 4B,C). In case of rat treated with TC extract, the liver histology was found to be identical to that of NC rats (Fig. 4D–F). These results indicate that treatment with TC protects the liver from inflammation-induced cellular damages.

**Figure 5:**
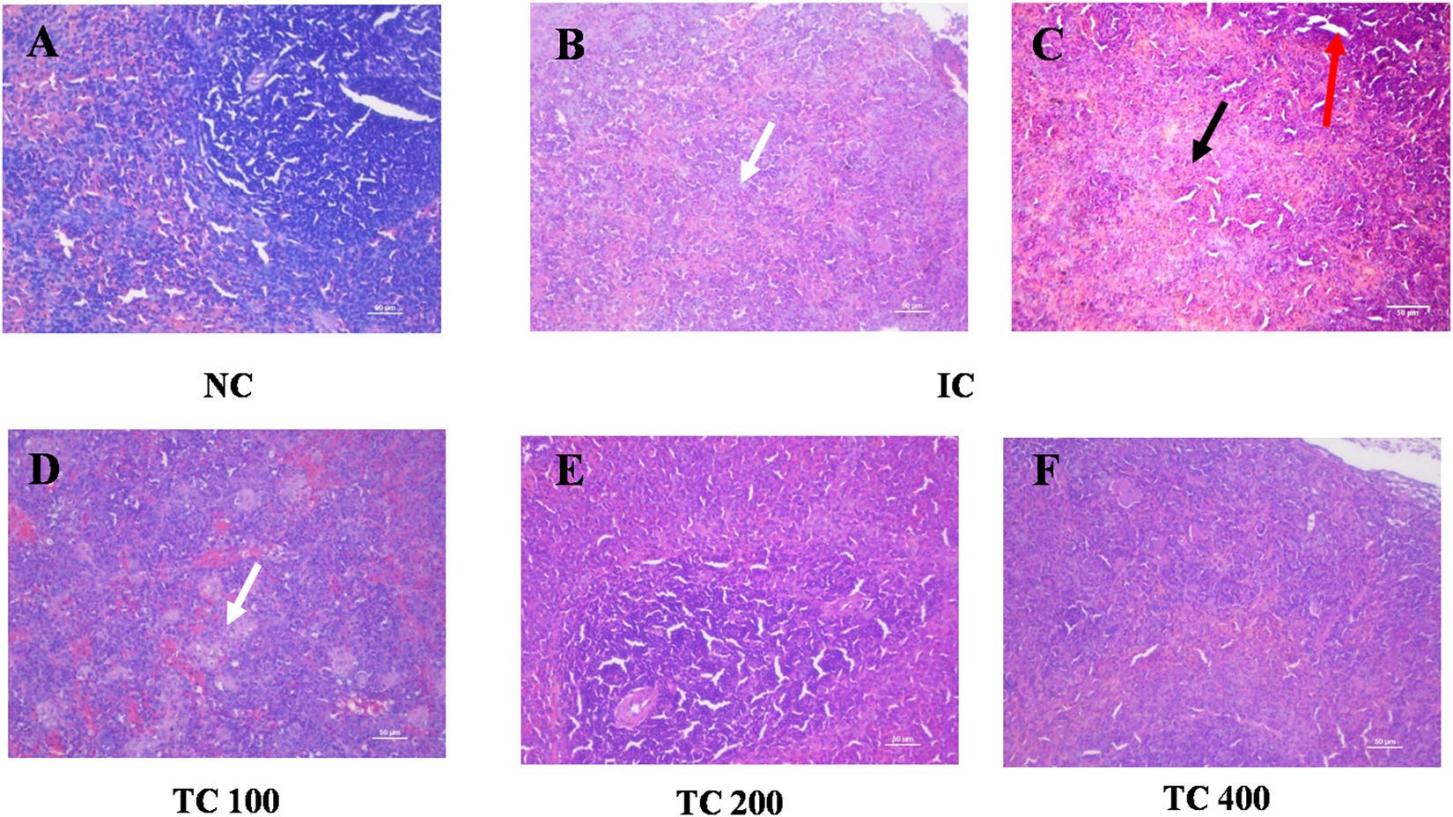
Protective effect of TC against infection-induced splenomegaly. (**A**–**F**) In the figure, white arrow shows depletion of white pulp, black arrow shows cellular rapture and structural changes, and red arrow shows necrotic white patches. NC: Normal control, IC: Inflammatory control, TC 100: 100 mg/kg, TC 200: 200 mg/kg, and TC 400: 400 mg/kg of body weight of TC aqueous extract. Number of animals per group = 5. Scale bar = 50 µm.

Similar to the liver, spleen also showed severe congestion, hemorrhagic changes with accumulation of RBCs in splenic parenchyma and, moderate hemosiderosis in splenic parenchyma in response to inflammation. Moreover, degenerative changes of cellular components and severe depletion of lymphocytes in white pulp was also evident (Fig. 5B,C). In the case of rats treated with TC, a reduction in accumulation of RBCs was observed. The treatment with TC was able to protect the spleen from inflammatory shock due to induction of inflammation. At 400 mg/kg body weight dose of TC, complete protection against HKBA-induced inflammation was observed (Fig. 5F).

### Gene expression studies

**Figure 6:**
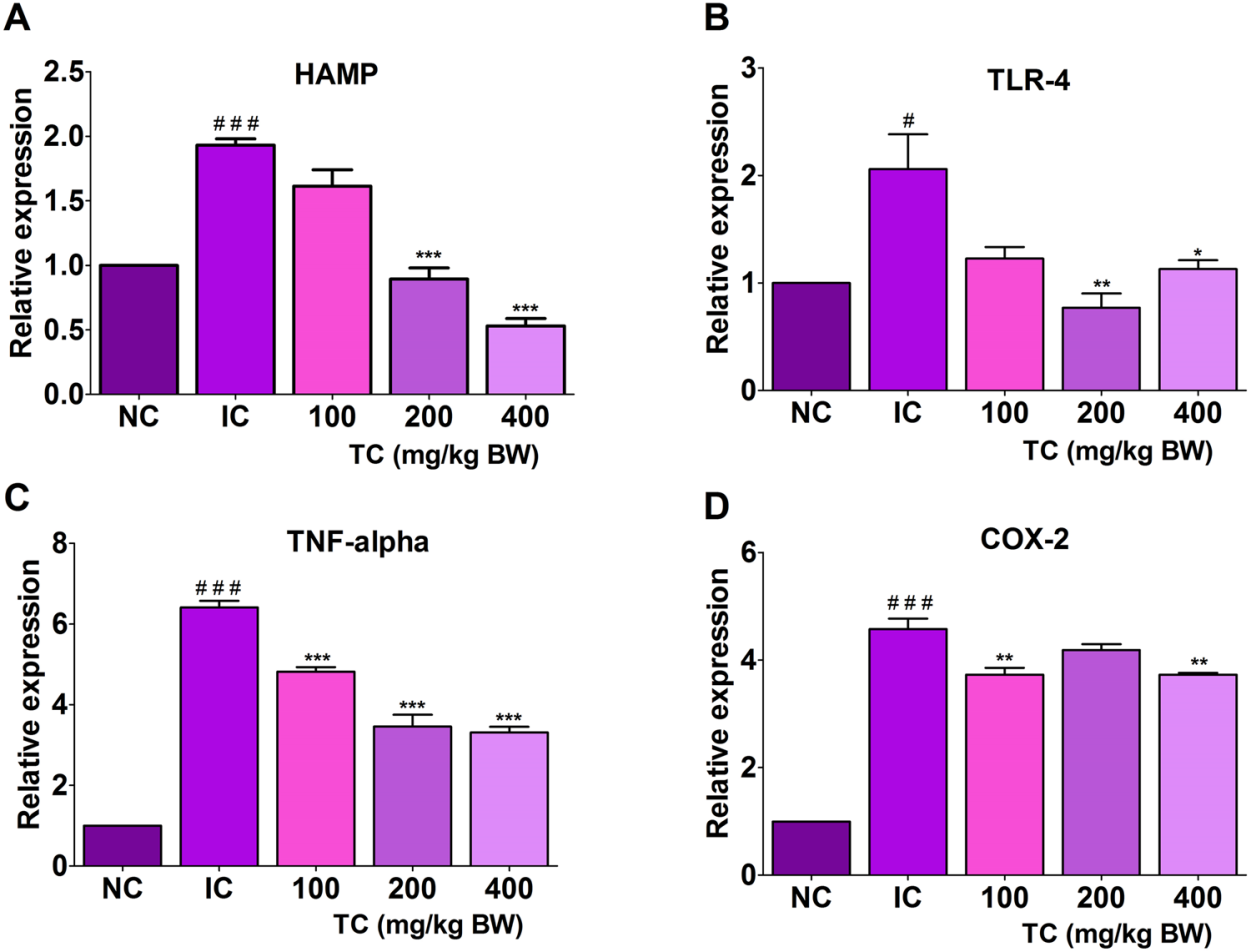
Effect of TC on hepatic expression of HAMP (**A**) TLR-4 (**B**) TNF-α (**C**) and COX-2 (**D**) genes. IC: Inflammatory control, 100: 100 mg/kg, 200: 200 mg/kg, and 400: 400 mg/kg of body weight of TC aqueous extract. Number of animals per group = 5. Results are shown as mean ± SEM. Statistical analyses were carried out by using one-way ANOVA and Bonferroni *post-hoc* test with *p < 0.05, **p < 0.01 and ***p < 0.001 against the IC group and ^#^*p* < 0.05, ^##^*p* < 0.01 and ^###^*p* < 0.001 against control.

The gene expression of human anti-microbial peptide (HAMP which encodes hepcidin), TLR-4, tumor necrosis factor-alpha (TNF-α), and cyclooxygenase-2 (Cox-2) genes was found to be significantly up-regulated in rats from the IC group as compared to NC group (Fig. 6A–D). A dose-dependent down-regulation in HAMP gene expression was observed in rats treated with TC as compared to IC group rats. Moreover, the relative expression of the HAMP gene was found lower in rats treated with TC at a dose of 200 and 400 mg/kg body weight than NC group rats (Fig. 6A). We further examined the changes in TLR-4 expression in rats. Rats treated with TC at a dose of 200 and 400 mg/kg body weight showed significant down-regulation in the expression of TLR-4 compared to NC group rats. Also, the relative expression of TLR-4 was found lower in rats treated with 200 mg/kg body weight of TC compared to control rats (Fig. 6D).

The treatment of TC in rats also affected the gene expression of TNF-α and Cox-2 as shown in Fig. 6C,D. Rats treated with TC showed significantly lower gene expression of TNF-α and Cox-2 as compared to IC group rats. However, this relative expression of TNF-α and Cox-2 genes in TC treated rats still higher than control rats. Overall results showed that treatment with TC showed anti-inflammatory action by down-regulating the TNF-α and Cox-2 genes expression and also inhibiting HAMP and TLR-4 gene expression. Overall *in vivo* studies suggest the protective effect of TC against AI via down-regulation of HAMP expression which maintains the optimum circulating iron levels.

### *In vitro* studies

**Figure 7:**
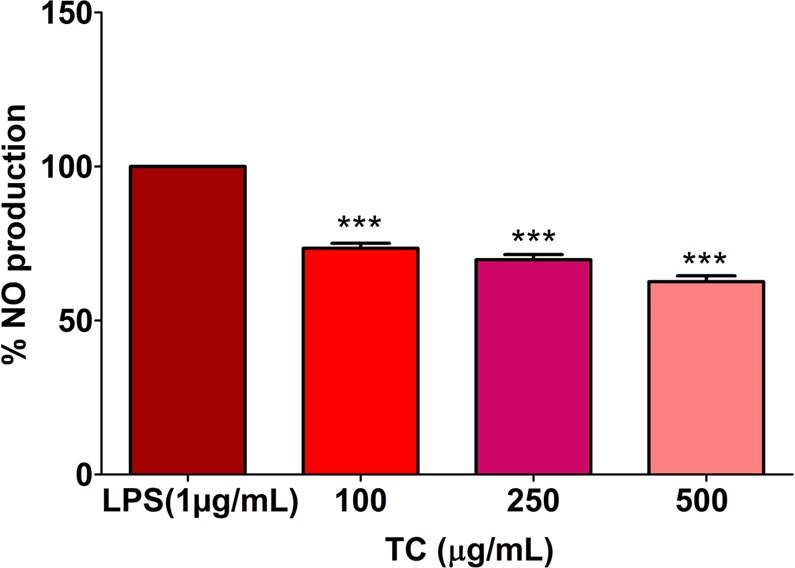
Effects of TC extract on NO production in RAW 264.7 cells. Data are expressed as the mean ± SEM of at least three independent experiments. Statistical analyses were carried out by using one-way ANOVA and Bonferroni post-hoc test with ***p < 0.001 against the LPS-stimulated positive control.

We first analyzed the effect of TC extract on LPS induced NO production. RAW 264.7 cells were pre-treated with TC extracts (100 to 500 µg/mL) then LPS was added in the medium to induce inflammation. The pretreatment of TC extract showed a significant reduction in NO production as compared to untreated cells (Fig. 7).

**Figure 8:**
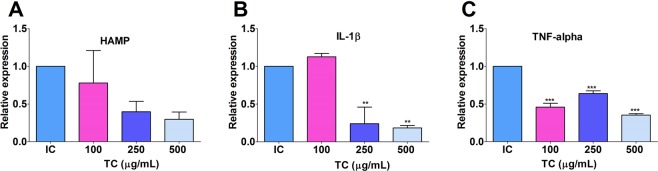
Effects of TC extract on LPS-induced mRNA expression of HAMP (**A**) TNF-α (**B**) IL-1β (**C**) genes in RAW264.7 cells. Results are in Means ± SEM are shown. IC: Cells treated with LPS (1 µg/mL). **p < 0.01 and ***p < 0.001 against the LPS-stimulated positive control.

RAW 264.7 cells pre-treated with TC extract (100 to 500 µg/mL) showed a down-regulation in gene expression of HAMP as compared to LPS stimulated untreated cells in dose-dependent manner (Fig. 8A). In the case of interleukin-1beta (IL-1β), the significant inhibitory effect of TC extract was observed only at higher concentration i.e. 250 and 500 µg/mL (Fig. 8B). Further, a significant down-regulation in the expression of TNF-α was also observed in cells treated with TC as compared to LPS stimulated untreated cells (Fig. 8C). Together, these findings indicated that TC extract may modulate the iron metabolism by inhibiting the expression of hepcidin as well as other inflammatory mediators.

### Quantification of tinosporaside

**Figure 9:**
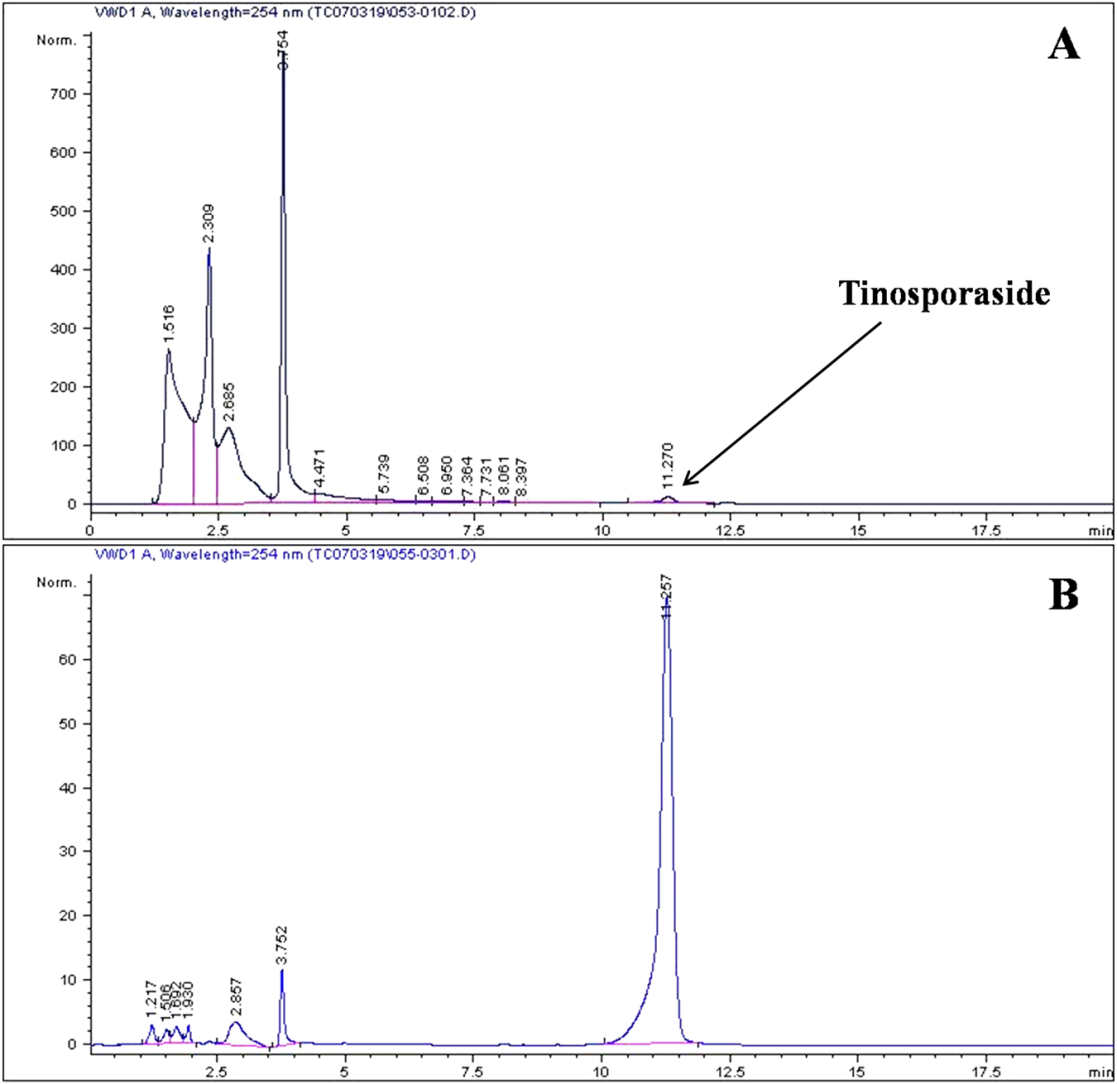
The chromatogram of TC extract showing  the presence of tinosporaside (**A**). The chromatogram of pure  tinosporaside (**B**).

Standardization of TC was carried out by HPLC under the isocratic conditions using the external standard calibration technique. Tinosporaside was used as a standard for analysis. Calibration curve was plotted by plotting peak areas Vs concentrations (ranges from 0.125, 0.250, 0.5, 1.0 and 2 mg/mL). Calibration plot shows a good linearity between concentrations and the peak area, with the correlation coefficient (*r*^2^) of 0.999. Chromatograms of the extract and their respective markers are shown in Fig. 9. Retention time (Rt) of tinosporaside was found to be 11.257 min. The amount of tinosporaside present in the TC extract was found to be 22.4 µg/mg of extract.

### Anti-inflammatory activity of tinosporaside

**Figure 10:**
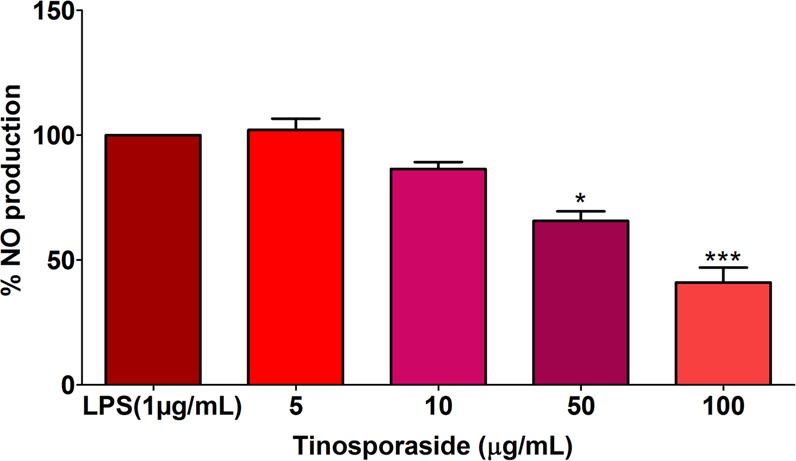
Effects of tinosporaside on NO production in RAW 264.7 cells. Data are expressed as the mean ± SEM of at least three independent experiments. Statistical analyses were carried out by using one-way ANOVA and Bonferroni post-hoc test with *p < 0.05, ***p < 0.001 against the LPS-stimulated positive control.

Anti-inflammatory potential of tinosporaside was carried out by estimating NO production in RAW 264.7 cells. RAW 264.7 cells were pre-treated with tinosporaside (5 to 100 µg/mL) then LPS was added in the medium to induce inflammation. A significant reduction in NO production was observed at a concentration 50 and 100 µg/mL as compared to untreated cells (Fig. 10).

## Discussion

In this study, we evaluated the efficacy of TC against AI using *in vivo* and *in vitro* models. AI model was developed in Wistar rats by repeated injections of HKBA showing a moderate degree of anemia. The HKBA-induced anemia was characterized by suppressed levels of Hb, low circulating iron levels along with reduced iron stores in liver and spleen and high inflammatory cytokine and hepcidin expression. TC treatment in rats showed improvement in Hb levels and RBC count. Moreover, a significant increased in the iron content of the serum, liver, and spleen was observed after TC treatment. It was also shown that TC treatment resulted in a significant decreased in gene expression of hepcidin and inflammatory biomarkers in the liver. Further our *in vitro* studies showed that treatment with TC in RAW 264.7 cells significantly reduced gene expression of inflammatory cytokines and hepcidin and also inhibited NO production stimulated with LPS. HPLC analysis showed the presence of tinosporaside and decreased in NO production in RAW 264.7 cells confirms its anti-inflammatory potential. All these findings suggest that the protective effects of TC extract could be due to its anti-inflammatory and anti-hepcidin action along with an improvement in overall iron status. To the best of our knowledge, this study is the first report on the protective effects of TC in an AI rat model.

The toxicity of TC was evaluated *in vivo*. No toxic effects were observed at the dose rate of 2000 mg/kg body weight. Thus, all the doses used in this study were safe and well below the doses used for toxicity studies. After confirming the nontoxic nature of TC, we studied the effect of TC in a rat model of AI. Several mouse models of inflammation have been developed for the testing of interventions in AI, including turpentine, LPS and CFA^10^. Sasu *et al*. (2010) reported AI model with a single i.p injection of HKBA which results in a nadir hemoglobin that is, 50% of the controls^11^. This mouse model shows the key features implicated in human AI, including hypoferremia and iron-restricted erythropoiesis with preserved iron stores, increased levels of inflammatory markers. Therefore on the basis of this background, we developed an AI model in Wistar rats using repeated injections of HKBA. Like a mouse model, we observed significantly decreased in Hb levels and RBC count in HKBA injected rats than control rats, indicating the development of anemia in rats. Therefore, we further tested the efficacy of TC against AI using this model. The rats treated with TC showed significant improvement in Hb levels and RBC count. Previous *in vivo* studies reported an enhancement in Hb levels and RBC count upon TC treatment^12,13^ in different animal models. Hence our findings confirm that TC treatments have beneficial effects on Hb levels and RBC count in AI rats.

To understand the possible mechanism of overall improvement in Hb levels and RBC count in TC treated rats, we further examined the effect of TC on serum and tissues iron levels in rats. HKBA-injected rats rapidly developed a hypoferremia compared with control rats. Serum iron levels were found to be significantly lower in HKBA-injected rats than control rats. Moreover, HKBA-injected rats had significantly reduced liver and spleen iron stores as compared to control rats. However, TC treated rats showed a significant improvement in serum as well as tissues iron levels. The liver maintains systemic iron homeostasis by regulating the levels of hepcidin as well as serves as a reservoir for excess iron. Splenic macrophages are involved in iron recycling from aged or damaged erythrocytes through the process of erythrophagocytosis^14^. The iron released form erythrophagocytosis is either stored in form ferritin or transported to other tissues via transferrin-mediated pathway^14^. Hence an improvement in the liver and spleen iron stores and circulating iron levels in TC treated rats indicate that iron status is a significant contributor to the suppression of anemia in rats.

Hepcidin is a liver-derived peptide which regulates iron metabolism under different conditions including inflammation^2^. Inflammation stimulates expression of hepcidin along with IL-1β and TNF-α leading to inhibition of iron absorption by enterocytes and iron recycling by macrophages. Thus hepcidin limits iron availability for the production of RBCs^15^. In our study, the repeated injection of HKBA resulted in higher hepcidin and TNF-α gene expression in rats. The increased expression of TNF-α is known to impair RBC production, often due to unavailability of iron^15^. The treatment with TC in rats showed a significantly lower hepatic hepcidin and TNF-α gene expression. Similarly, the low expression of hepcidin, TNF-α, and IL-1β genes along with decreased NO production was observed in TC pre-treated RAW 264.7 cells compared to untreated cells.

Several studies reported the role of TLRs in the regulation of iron metabolism^16,17^. As the host immune response restricts iron availability to invading pathogens, hepcidin is induced by inflammatory cytokines that are produced by macrophages expressing TLRs. Here in our study, we found that TLR-4 gene expression in the liver of HKBA injected rats was significantly up-regulated than control rats. A recent study reported the LPS-induced hepcidin expression in hepatocytes through the TLR-4 pathway^17^. The study found that LPS-induced hepcidin expression by hepatocytes is regulated by its specific receptor, TLR-4, via a MyD88-dependent signaling pathway. The treatment with TC in rats caused a significant reduction in hepatic TLR-4 expression. Therefore, the down-regulation of hepcidin expression in TC treated rats may be due to the reduction in the expression of TLR-4. However, further studies are required to elucidate the exact mechanism of TC mediated regulation of hepcidin via TLR-4. Additionally, it was found that the hepcidin is regulated in microglial cells through inhibition of Cox-2 expression^18^. Therefore TC may exert its anti-inflammatory action by inhibiting Cox-2 expression which may further down-regulates the hepcidin expression in liver.

Our *in vivo* studies were supported by the *in vitro* experiments using an aqueous extract of TC on RAW 264.7 cells. The cells pre-treated with TC extract showed a reduction in the expression of hepcidin, TNF-α, IL-1β, genes and also inhibited production of NO. It is also known that under the inflammatory response, murine macrophages produce pro-inflammatory cytokines such as IL-1β, IL-6, and TNF-α^8^. Overall our findings suggest that TC extract has anti-inflammatory properties and also inhibits hepcidin expression.

The protective effect of TC on HKBA induced-inflammation was also evident from histological studies. The liver from HKBA injected rats showed degenerative changes including hepatocytes swelling and necrosis due to chronic inflammation. The liver histology of the animals pre-treated with TC exhibit improvement over HKBA injected rats in a dose-dependent manner. In addition, transaminase levels in the serum indicated protection against liver damage caused by inflammation at a higher dose of TC (Supplementary Table S1). The protection of liver histology by TC extracts have been reported previously^19^ in different rat models. Similar to the liver, the treatment with TC was able to protect the spleen from degenerative changes due to induction of inflammation. Previously, Sachdeva *et al*. 2014 reported protection of spleen histology by TC extract in combination with cisplatin in *Leishmania donovani* infected BALB/c mice^20^.

HPLC analysis showed the occurrence of tinosporaside in TC. Tinosporaside is a 18-norclerodane glucoside which is present in the stem bark of TC. The anti-inflammatory potential of tinosporaside has not been previously reported. In our study we found that tinosporaside inhibited NO production in dose dependent manner. Previous investigations described TC has numerous secondary metabolites. One of the contributing factors responsible for the anti-inflammatory effect of TC plant would be due to the combined or individual potential of the phytoconstituents present in this plant.

In conclusion, HKBA induces a moderate degree of anemia and chronic inflammation in Wistar rats. This model is characterized by reduced circulating iron levels and diminished tissues iron and, elevated expression of inflammatory cytokines and hepcidin. The treatment with TC extract protects rats by improving iron status. This effect was chiefly associated with lowered expression of hepcidin. Consistent with *in vivo* results, our *in vitro* studies with TC aqueous extract showed that TC could inhibit LPS-induced up-regulation of inflammatory genes (TNF-α, IL-1β) and NO levels in RAW 264.7 cells. Further studies are ongoing in various systems to identify the underlying biomolecular mechanisms for anti-inflammatory activity of TC as well as tinosporaside to obtain an effective treatment for AI.

## Materials and Methods

Sodium acetate, guanidine hydrochloride, and glacial acetic acid were purchased from SRL, India. Lipopolysaccharide (LPS), Carboxy Methyl Cellulose (CMC), Tinosporaside, Ferrozine (3-(2-Pyridyl)-5,6-diphenyl-1,2,4-triazine-p,p′-di-sulfonic acid monosodium salt hydrate)^®^ and, Complete Freund’s Adjuvant (CFA) were purchased from Sigma-Aldrich, India.

### Authentication of TC extract and quantification of tinosporaside

The aqueous extract of TC was purchased from Chaitanya Pharmaceuticals Pvt. Ltd., India. The extract was authenticated from the Drug and Plant authentication service center, Agharkar Research Institute, Pune, India. The authentication of the extract was carried out using Tinosporaside as an internal standard using the HPTLC method^21^. To quantify tinosporaside in TC extract we performed high performance liquid chromatography (HPLC) analysis using Agilent 1100 series quaternary HPLC system. A Lichro CART Purosphere STAR RP-18e (Merck Darmstadt, Germany) (250 mm × 4.6 mm, 5 μm particle size) was used. Tinosporaside, was quantified in the extract at 25 °C, the analysis was performed at a flow rate of 1.0 mL/min using mobile phase consisted of water: acetonitrile (75:25 v/v). The variable wavelength detector was set at wavelength of 254 nm for quantification. The tinosporaside in the extract were quantified using the calibration curve^22^.

### Animals

The animal studies were carried out in accordance with the recommendations and approval of the Institutional Animal Ethical Committee of Agharkar Research Institute, Pune, India (ARI/IAEC/2015/01). Male Wistar rats 6–8 week old (180–200 gm) were used in this study. Inbred Wistar strain of rats was housed at standard environmental conditions. They were fed on standard chow and provided purified water *ad libitum*.

### Acute toxicity

The acute oral toxicity studies were performed in Wistar rats to assess the safety of TC using revised Organization for Economic Co-operation and Development (OECD)-423 guidelines^23^. This study was initiated with the limit test at a single highest dose of 2000 mg/kg body weight. The animals were grouped into control and treated groups (n = 3). The TC extract was suspended in 0.5% CMC and administrated orally with a single dose of 2000 mg/kg body weight. The control group animals received an equivalent quantity of 0.5% CMC without extract. All the animals were kept under observations for 14 days and their body weight was recorded at weekly interval. On the 14^th^ day, animals were sacrificed and all organs were collected.

### Experimental design

**Figure 11:**
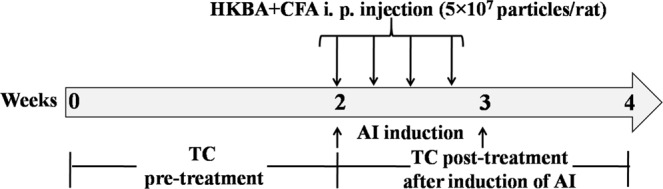
Experimental timeline diagram indicating various stages involved in this study.

Animals were divided into 5 groups namely normal control (NC), inflammatory control (IC) and three different treatment groups receiving TC at dose rates of 100, 200 or 400 mg/kg of body weight. Each group contains six animals. TC was administered orally suspended in 0.5% CMC once a day at fix time for two weeks prior to induction of AI (Fig. 11). Rats from NC and IC groups received 0.5% CMC. To induce AI, heat-killed *Brucella abortus* antigen (HKBA, Indian Immunologicals Limited, India) was injected intraperitoneally (i.p.) for four times on every alternate day at a dose of 5 × 10^7^ particles/rat emulsified along with 0.4 mL CFA except the rats from NC groups which received saline instead of HKBA. Treatment of TC was continued further for 2 weeks after HKBA injections. Blood was withdrawn weekly to measure hemoglobin (Hb), total red blood cells (RBC) count, and serum iron levels. Rats were sacrificed at the end of the study to collect liver and spleen.

### Biochemical and histological analysis

The Hb concentrations were measured using cyanmethaemoglobin method^24^. RBC count was carried out by standard laboratory procedure with a hemocytometer^25^. Iron contents of serum, liver, and spleen were measured spectrophotometrically at 562 nm using ferrozine assay, as described previously with some modifications^26^. The serum transaminase levels were analyzed by an automated analyzer using diagnostic kits (ERBA, Czech Republic). The histology was performed using standard protocol and the tissues were stained using hematoxylin and eosin (H&E).

### Nitric oxide estimation

The mouse macrophage RAW 264.7 cell line was purchased from National Centre for Cell Science, Pune, India. Cells were cultured and maintained in DMEM with 10% heat-inactivated FBS, penicillin (100 U/mL), and streptomycin (100 µg/mL) in a humidified atmosphere with 5% CO_2_ at 37 °C. Experiments were carried out at the density of 1 × 10^5^ cells/mL. The pre-treatments of TC extracts (100–500 µg/mL) and tinosporaside (5–100 µg/mL) were given to cells for 4 h at 37 °C. After pre-treatment, cells were stimulated with LPS (1 µg/mL) for further 20 h. The levels of nitrite in cell culture media were determined using commercial NO detection kit (Sigma Aldrich, India). The cell viability of the LPS-treated group was expressed as 100%.

### qRT-PCR analysis

Real-time detection of PCR was performed using PCRmax Eco 48 system (PCRmax, UK). Total RNA was extracted from RAW 264.7 cells and frozen liver tissues using TRIzol reagent (Thermo Fisher Scientific, India) according to the manufacturer’s protocol. One microgram of the RNA obtained was used to generate cDNA following the protocol for High-Capacity cDNA Reverse Transcription Kit (Applied Biosystems™, Thermo Fisher Scientific, India). The obtained cDNA was amplified using SYBR Green Master Mix kit Kit (Applied Biosystems™, Thermo Fisher Scientific, India). The primers used for the study are listed in Table 3. All reactions were carried out in triplicate. The relative expressions of the various genes of interest were determined in the control, inflammatory control and TC treated groups by normalization to Glyceraldehyde 3-phosphate dehydrogenase (GAPDH: for RAW 264.7 cells) and β-actin (for rat liver) genes.

**Table 3:** Primers used for qRT-PCR assays.

Gene	Forward (5′ → 3′)	Reverse (5′ → 3′)
**RAW 264.7 cells**		
TNF-α	GACCCTCACACTCAGATCATCTTCT	CCACTTGGTGGTTTGCTACGA
HAMP	GCAGAAGAGAAGGAAGAGAGACACC	TGTAGAGAGGTCAGGATGTGGCTC
IL-1β	AAATACCTGTGGCCTTGGGC	CTTGGGATCCACACTCTCCAG
GAPDH	GTGTGAACGGATTTGGCCGTATTGGGCG	TCGCTCCTGGAAGATGGTGATGGGC
**Wistar rats**		
TNF-α	CCCAGACCCTCACACTCAGATCAT	GCAGCCTTGTCCCTTGAAGAGAA
COX-2	CGGAGGAGAAGTGGGGTTTAGGAT	TGGGAGGCACTTGCGTTGATGG
HAMP	GAAGGCAAGATGGCACTAAGC	CAGAGCCGTAGTCTGTCTCG
TLR-4	AGCCGGAAAGTTATTGTGGTGGTG	CAACGGCTCTGGATAAAGTGTCT
β-actin	GGATCTACCAGTCTAACA	GATAGTTAGTGATCCCACTG

TNF-α: Tumor necrosis factor-alpha, HAMP: Human antimicrobial peptide, IL-1β: Interleukin-1beta, GAPDH: Glyceraldehyde 3-phosphate dehydrogenase, COX-2: Cyclooxygenase-2, TLR-4: Tall like receptor-4.

### Statistical analysis

All statistical analyses were performed by using Statistical Package for Social Sciences (SPSS for windows version 17.0, SPSS Inc., Chicago, US). Values are expressed as a mean ± standard error of the mean (SEM). Data were analyzed using ANOVA and multiple comparisons were carried out using the Bonferroni *post-hoc* test. Statistical significance was considered for *p* values < 0.05.

## Supplementary information

Tinospora cordifolia protects against inflammation associated anemia by modulating inflammatory cytokines and hepcidin expression in male Wistar rats

## Data Availability

The datasets generated during and/or analyzed during the current study are available from the corresponding author on reasonable request.
