# Supplementary material for: Tinospora cordifolia protects against inflammation associated anemia by modulating inflammatory cytokines and hepcidin expression in male Wistar rats
Source: Sci Rep. 2019 Jul 29;9:10969. doi: 10.1038/s41598-019-47458-0 (PMC6662690; doi:10.1038/s41598-019-47458-0)
Supplement: Supplementary file 1 — Tinospora cordifolia protects against inflammation associated anemia by modulating inflammatory cytokines and hepcidin expression in male Wistar rats [file 41598_2019_47458_MOESM1_ESM.doc]

**SUPPORTING INFORMATION**

***Tinospora cordifolia* protects against inflammation associated anemia by modulating inflammatory cytokines and hepcidin expression in male Wistar rats**

Niraj S. Ghatpande, Ashwini. V. Misar , Ravindra, J. Waghole, Sachin H. Jadhav , and Prasad. P. Kulkarni^*^

**Supplementary Table 1. Changes in SGOT and SGPT levels in rats of different groups.**

| **Parameters** | **Control** | **Inflammatory control** | **G100** | **G200** | **G400** |
| --- | --- | --- | --- | --- | --- |
| **SGOT (IU/L)** | 20.6 ± 1.5 | 34.60 ± 2.6^#^ | 32.4 ± 4.3 | 17.4 ± 1.2^**^ | 16.6 ± 1.9^***^ |
| **SGPT (IU/L)** | 20.8 ± 2.2 | 44.0 ± 3.0^###^ | 27.0 ± 3.5^*^ | 36.2 ± 4.4 | 25.8 ± 2.5^**^ |

SGOT:AST: Aspartate aminotransferase (AST) and SGPT:ALT: Alanine transaminase. IC: Inflammatory control, 100: 100 mg/kg, 200: 200 mg/kg, and 400: 400 mg/kg of body weight of TC aqueous extract. Results are shown as mean ± SEM. Statistical analyses were carried out by using one-way ANOVA and Bonferroni *post-hoc*  test with *: p < 0.05, **: p < 0.01 and ***: p < 0.001 against the IC group and #: p < 0.05, ##: p < 0.01 and ###:p < 0.001 against control.
